# Influence of age, sex, body mass index, alcohol, and smoking on shear wave velocity (p-SWE) of the pancreas

**DOI:** 10.1007/s00261-016-0661-7

**Published:** 2016-02-15

**Authors:** Sabina Stumpf, Heike Jaeger, Tilmann Graeter, Suemeyra Oeztuerk, Julian Schmidberger, Mark Martin Haenle, Wolfgang Kratzer, The Elasto-Study Group Ulm

**Affiliations:** Department of Internal Medicine I, University Hospital Ulm, Albert-Einstein-Allee 23, 89081 Ulm, Germany; Division of Neurophysiology, FASCIA Research, Albert-Einstein-Allee 11, 89081 Ulm, Germany; Department of Diagnostic and Interventional Radiology, University Hospital Ulm, Albert-Einstein-Allee 23, 89081 Ulm, Germany

**Keywords:** Pancreas, ARFI, VTQ, Influencing factors, Standard values, p-SWE

## Abstract

**Purpose:**

A variety of elastographic techniques have been developed to facilitate the non-invasive assessment of tissue properties. The goal of the study was to examine the influence of gender, age, BMI, alcohol consumption, and smoking in healthy volunteers.

**Methods:**

Of the 263 participants who met all the study inclusion criteria, 234 had successful measurements. The examination was performed with the Siemens Acuson S3000 (Siemens Healthcare, Erlangen, Germany), using the 6C1 curved array transducer with the virtual touch tissue quantification (VTQ) method.

**Results:**

The values determined with the curved array in the head of the pancreas were 1.44 ± 0.39 m/s for women and 1.19 ± 0.29 m/s for men; in the body, the results were 1.49 ± 0.37 m/s for women and 1.26 ± 0.30  m/s for men; in the tail, the corresponding values were 1.29 ± 0.36 m/s for women and 1.05 ± 0.30 m/s for men. Comparison of gender showed that men have significantly lower mean values than women. There were significantly higher values in all parts of the organ with the increasing age of the participants (*p* < 0.0001). For BMI, there was a significant correlation with the values only when considering the BMI in continuous form. Alcohol consumption and smoking did not have any significant effects.

**Conclusions:**

ARFI-VTQ is qualified for use on pancreatic tissue. Further studies are required to examine the influence of other factors in larger populations.

The incidence of pancreatic disease, such as cancer, cystic lesions, and acute and chronic pancreatitis, is rising [1–3]. Early diagnosis of these diseases is extremely important, especially in the case of pancreatic cancer [4]. Computed tomography (CT), magnetic resonance imaging (MRI), endoscopic ultrasound (EUS), positron emission tomography (PET), PET-CT, and transabdominal ultrasound scanning (TUS) are currently available imaging techniques for diagnostic purposes. Various elastographic procedures have also been available for several years [5].

Elastographic procedures allow us to determine the stiffness of tissues. At the present time, the available techniques are strain elastography, which demonstrates the tissue elasticity by external compression, and point shear wave elastography (p-SWE), single shot (VTIQ), 2D-SWE, and real-time (SSI technology). A recent study shows that the various elastography methods seem to be comparable [6]. Elastographic methods have been used most extensively to investigate diffuse liver disease, and breast and thyroid tissues [6–12], while there are only limited data on transabdominal examination of the pancreas. The findings of earlier transabdominal elastographic studies to detect acute or chronic pancreatitis are still conflicting [13–15]. More recent investigations to determine the degree of pancreatic fibrosis show promising results, however, also in the assessment of patients with cystic fibrosis [16, 17]. Two recent studies to detect solid pancreatic masses found clearly higher shear wave velocity in solid tumors of the pancreas than in the surrounding parenchymal tissue [18, 19]. The value of pancreatic elastography in the assessment of cystic space-occupying lesions cannot be determined conclusively at the present time, as all the data come from just one research team [20]. The great value of endoscopic ultrasound (EUS) elastography in the investigation of the pancreas has been shown in recent studies [21]. Compared with transabdominal elastographic procedures, however, EUS procedures are more invasive and technically more demanding [19].

Systematic investigations of the influence of factors such as gender, age, body mass index (BMI), anatomical conditions, transducer frequency, respiration, and nutritional status on pancreatic measurements are somewhat limited [22–24]. Arda et al. found no differences in the pancreas due to gender in 127 subjects [24]. Gallotti et al. measured higher shear wave velocities in the head of the pancreas than in the body [22]. In their recent study on 210 healthy volunteers, Xie et al. did not find any effects of age, gender, BMI, organ size, or waist circumference [15]. Various other studies have examined healthy volunteers using the virtual touch tissue quantification (VTQ) technology from Siemens (Acuson S2000, Siemens Medical Solutions, Mountain View, CA, USA) [13, 22]. Yashima et al. studied 52 healthy volunteers and determined values of 1.23 ± 0.34, 1.30 ± 0.34, and 1.24 ± 0.5 m/s for the head, body, and tail of the pancreas, respectively [13]. Mateen et al. published a normal value of 1.27 ± 0.29 m/s for pancreatic tissue, likewise in 52 healthy volunteers, although they made no distinction between the head, body, and tail regions [14]. Gallotti et al. examined 35 patients and obtained a mean value of 1.40 m/s, but they also did not distinguish the different anatomical regions of the pancreas [22].

The aim of our study was therefore to investigate the influence of age, gender, BMI, alcohol consumption, and smoking on the shear wave velocity of the pancreas in healthy volunteers.

## Materials and methods

### Volunteers

The study was performed in accordance with the Helsinki Declaration and approved by the local ethics committee [25]. All subjects gave their written consent to participate in the study. We enrolled 324 healthy volunteers between March and August 2013. Healthy volunteers were defined as follows: persons without known pre-existing conditions, no drug revenues, physical and mental health at the time of the investigation. The medical history taken from each subject included previous illnesses, medications, and drug and alcohol consumption. Subjects who admitted to drinking alcohol more than three times a week were allocated to the positive control group in this study. The random sample included only those subjects in whom we were able to obtain elastographic measurements for all three regions of the pancreas—head, body, and tail. Of the 324 healthy volunteers enrolled, we excluded 90 from the analysis for the following reasons: incomplete measurements, medication, drug consumption, chronic disease, BMI >30 kg/m^2^, and a history of cancer.

### Interview and questionnaire

Each interview was conducted by a trained interviewer. The standardized questionnaire included personal data (i.e., date of birth, gender, current weight, body size), health and social behavior (i.e., alcohol consumption), and other risk factors for medical diseases (i.e., smoking) and previous medical records of participants. For estimation of alcohol consumption and smoke behavior, each subject was asked if they drink alcohol and if they smoke.

### Elastography

**Fig. 1:**
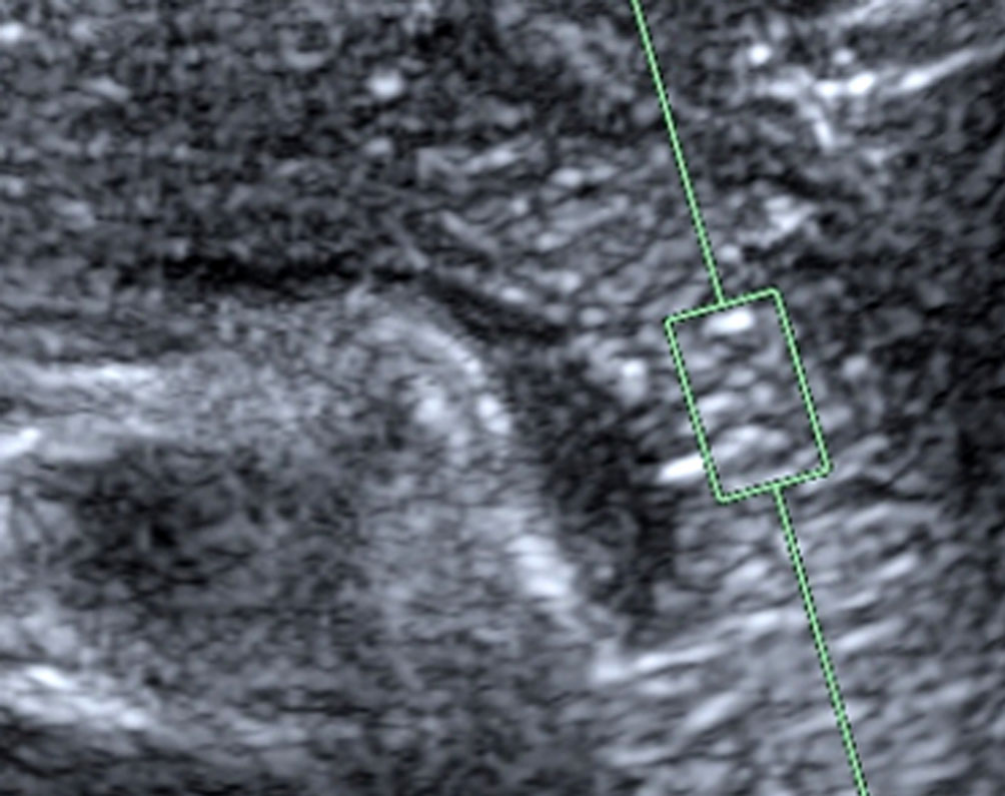
Measurement with the VTQ method

**Fig. 2:**
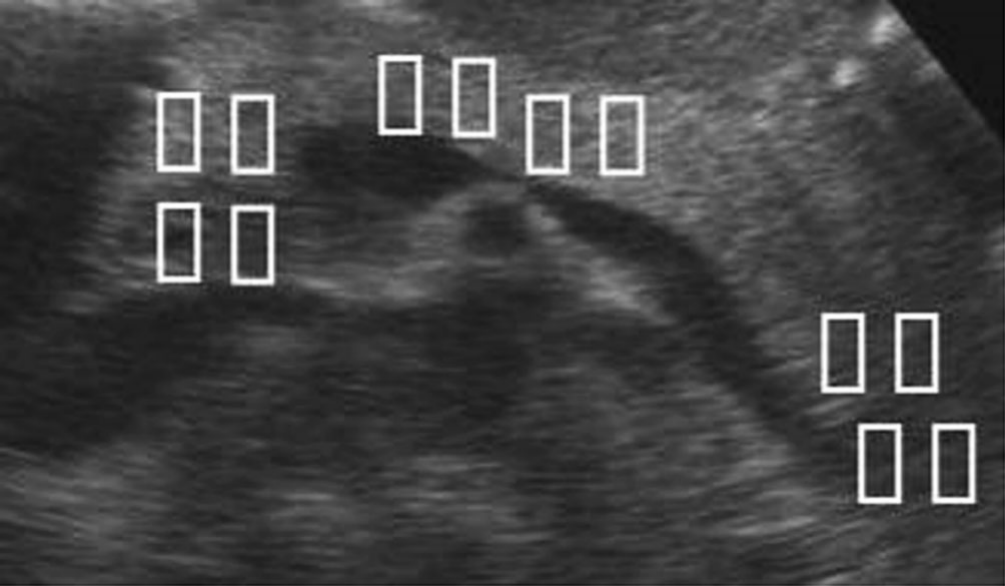
Sites of VTQ measurement

Measurements were made with a Siemens Acuson S3000 (Siemens Medical Solutions, Mountain View, CA, USA) and a 6C1 curved array transducer for VTQ (Fig. 1). To begin with, the pancreas was demonstrated in B-mode imaging. The echogenicity of the pancreas was then compared with the kidney/liver parenchyma. A diagnosis of pancreatic lipomatosis was made if the pancreas was clearly more echogenic than the liver parenchyma [26]. Patients fasted for 3 h prior to the examinations, which were then carried out with the patient lying down. In each case, we took four measurements in each of the head, body, and tail regions. Figure 2 shows the procedure for positioning the region of interest (ROI). So that we could obtain measurements in mid-respiration, the subject was instructed to breathe in deeply, breathe out, and then breathe normally, until told to stop breathing while the measurements were taken. This procedure prevented movement artifacts, as far as possible. We excluded subjects in whom we could not get clear views of the head, body, and tail of the pancreas, as well as all those with fewer than four measurements per region. The shear wave velocities for each measurement position were recorded in m/s.

### Reliability measurements

As nine examiners were involved in the study, they performed reliability measurements on phantoms to determine the inter-observer reproducibility. We selected four phantoms—a pig’s liver, pig’s muscle, and two balls (one of a hard and one of a soft material consistency)—and encased them in agarose gel. This allowed us to determine the intra- and inter-rater reliability.

### Statistical analyses

SPSS statistics software (IBM SPSS Statistics, Version 21) was used for the analyses. Mean, standard deviation (STD), median, and range were determined for continuous variables. Continuous variables of two or more independent groups were compared with Student’s *t* test or one-way ANOVA. Correlation between parameters were expressed with Pearson’s correlation coefficients. Parameters that were significant in univariate analysis (age, gender, BMI) were included in multivariate analysis using linear regression. The intra- and inter-rater reliability of measurements obtained with the curved array on the phantoms was checked with the intra-class correlation coefficient (ICC, two-factorial with the inclusion of phantom and mass effects). With this procedure, agreement is considered excellent with a figure of 0.75 or more, good with 0.60–0.74, moderate with 0.40–0.59, and poor with results of less than 0.40. We used two-tailed statistical tests to demonstrate all relationships and differences in the mean.

## Results

The sample consisted of 148 women (63.3%) and 86 men (36.8%). The mean age of the men was 31.5 ± 13.7 years, and that of the women 36.3 ± 15.5 years; the mean BMI was 22.5 ± 2.4 kg/m^2^. Overall, 9.9% of participants (*n* = 23) were smokers, while 11.1% of these healthy volunteers (*n* = 26) admitted that they drank alcohol more than three times a week.

The shear wave velocity obtained for the pancreas with the 6C1 curved array transducer was 1.35 ± 0.37 m/s for the head, 1.41 ± 0.36 m/s for the body, and 1.20 ± 0.36 m/s for the tail (Table 1). Independent of the location, the following values were determined: 1.31 ± 0.29 m/s (range 0.74–2.29). The values of the individual segments correlate significantly with each other ((head vs. body: *p* ≤ 0.0001 (*r* = 0.49120); head vs. tail: <0.0001 (*r* = 0.35442); body vs. tail: <0.0001 (*r* = 0.44167)). Dividing the subjects into three age groups (<25, 25–50, >50 years), however, the values for all three pancreatic regions differ significantly between the age groups. Measured values rose with increasing age (Table 2). In all three organ segments, the mean values for men were lower than those for women (*p* < 0.0001) (Table 3). In order to determine the effects of BMI on the measurements, we divided the healthy volunteers into two groups (BMI < 25 and BMI > 25). Comparing the results, it became apparent that the values does not differ between the two groups for all segments (head *p* = 0.4190; body *p* = 0.1806; tail *p* = 0.2952). There were no significant differences of the means between smokers (*n* = 23) and non-smokers (*n* = 211) (head: *p* = 0.4105; body: *p* = 0.4561; tail: *p* = 0.2624). In participants with a higher alcohol consumption (*n* = 26), there was a tendency toward higher values but the differences were not significant (head: *p* = 0.3690; body: *p* = 0.2131; tail: *p* = 0.1120).

**Table 1:** Shear wave velocity in relation to site of measurement, 6C1 curved array transducer with VTQ method (*n* = 234)

	Mean	95% CI		STD	Med	Min	Max
		LL	UL				
Head	1.35	1.30	1.39	0.37	1.28	0.70	2.73
Body	1.41	1.36	1.45	0.36	1.34	0.68	2.90
Tail	1.20	1.16	1.25	0.36	1.12	0.64	2.61

STD, standard deviation; Med, median; Min, minimum; Max, maximum; LL, lower limit; UL, upper limit; 95% CI, 95% confidence interval

**Table 2:** Results with the curved array transducer depending on age

	Mean ± STD (range)			*p* value
	Age < 25 (*n* = 84)	Age 25–50 (*n* = 105)	Age > 50 (*n* = 45)	
Head	1.27 ± 0.31 (0.76–2.33)	1.33 ± 0.38 (0.70–2.49)	1.53 ± 0.39 (0.80–2.73)	0.0004
Body	1.32 ± 0.33 (0.68–2.82)	1.40 ± 0.33 (0.84–2.58)	1.59 ± 0.43 (0.98–2.90)	0.0002
Tail	1.10 ± 0.29 (0.70–2.12)	1.16 ± 0.32 (0.64–2.35)	1.49 ± 0.41 (0.86–2.61)	<0.0001

**Table 3:** Results according to gender

	MW	STD	min	max	*t*	*p*
Head						
Women	1.44	0.39	0.86	2.73	5.024	<0.000
Men	1.19	0.29	0.70	2.17		
Body						
Women	1.49	0.37	0.85	2.90	5.375	<0.000
Men	1.26	0.30	0.68	2.82		
Tail						
Women	1.29	0.36	0.73	2.61	5.303	<0.000
Men	1.05	0.30	0.64	2.16		

M, mean; STD, standard deviation; min, minimum; max, maximum

The results of the multivariate analysis show for the areas head and body a significant association between shear wave velocity and age (continuous), BMI (continuous), and gender. For the section tail, the same result could be shown like for the other two sections, only BMI had no influence on the shear wave values (Table 4).

**Table 4:** Factors associated with the shearwave velocities

Factor	Univariate analysis		Multivariate analysis		
	Estimate	*p*	Estimate	*p*	95% confidence interval
Head					
Gender	−0.24741	<0.0001	−0.17241	0.0005	−0.26782 to −0.07699
BMI	−0.02510	0.0131	0.00773	0.0011	−0.05408 to −0.01363
Age	0.000672	<0.0001	−0.03386	<0.0001	0.00455 to 0.01091
Body					
Gender	−0.23797	<0.0001	−0.15052	0.0015	−0.24268 to −0.05836
BMI	−0.03517	0.0003	−0.04491	<0.0001	−0.06444 to −0.02537
Age	0.00600	0.0001	0.00772	<0.0001	0.00465 – 0.01079
Tail					
Gender	−0.23548	<0.0001	−0.17683	0.0001	−0.26612 to −0.08754
BMI	0.00353	0.7217	−0.00989	0.3042	−0.02882 to 0.00903
Age	0.01031	<0.0001	0.00999	<0.0001	0.00707 to 0.01296

The ICCs for the intra-rater reliability of the individual examiners ranged from 0.857 to 0.979, indicating excellent reliability (Table 5). The inter-rater reliability was also excellent, with figures of 0.931 in round 1 and 0.898 in round 2 (Table 6).

**Table 5:** Results of intra-rater reliability

Examiner	ICC	95% CI	
		LL	UL
1	0.943	0.842	0.986
2	0.885	0.701	0.970
3	0.964	0.897	0.991
4	0.979	0.938	0.995
5	0.979	0.940	0.995
6	0.920	0.783	0.979
7	0.882	0.693	0.969
8	0.857	0.638	0.962
9	0.860	0.643	0.963
Transducer mounted on a tripod	0.982	0.948	0.996

ICC, intra-class correlation; CI, confidence interval; LL, lower limit; UL, upper limit

**Table 6:** Results of inter-reliability measurements according to round of measurements

	ICC	95% CI	
		LL	UL
Round 1	0.931	0.848	0.981
Round 2	0.898	0.751	0.982

ICC, intra-class correlation; 95% CI, 95% confidence interval; LL, lower limit; UL, upper limit

## Discussion

To the best of our knowledge, the present study is the largest one investigating the factors affecting measurements of the pancreas with a Siemens Acuson S3000. Our data confirm earlier results obtained using the VTQ technique with an Acuson S2000. Our findings also confirm data from Yashima and Gallotti, which showed different shear wave velocities for the head, body, and tail of the pancreas [13, 22]. The reason for the different measurements obtained from these anatomical regions may be different histological properties or differences in the measurement positions imposed by the anatomy when determining shear wave velocity. One possible explanation is the different angles of the ROIs when measuring the head and tail, as there is deviation from the vertical in the measurements [27]. Another explanation might be the deeper location resulting in lower measurements from the head and tail in comparison with the body of the pancreas.

Our results show a significant difference between the genders. In a further study from our research group, a gender difference could also be shown on the liver [28]. A deep dependence of the shear wave velocity was detected by various working groups [28, 29]. One possible explanation for the different values could be the different organ position in men and women. Due to a thicker muscle layer, the organ in males is usually farther from the transducer away than in women. The difference between the measurement results could therefore be due to measuring technique-related error.

Our data show a significant relationship between the increase in the density of the pancreatic tissue and age. In 202 children, Lee et al. also demonstrated an age-dependent increase in shear wave velocity [30]. Contrary to these results, Xie et al. found that age had no effect on shear wave velocity in 210 healthy volunteers [15]. The influence of the factor “age” has not yet been adequately studied. Two studies see a correlation between higher shear wave velocity and age [31, 32]. Elastographic examinations on the kidneys have confirmed a significant age-dependent increase in the velocity in children [33]. Age-related remodeling processes within organs may explain these changes. With increasing age, the tissue becomes harder due to inflammation, atrophy, or fibrotic changes and the associated loss of elasticity leads to higher shear wave velocities. Various research teams have shown gender-specific differences [32–34]. On the other hand, the results from Xie et al. refute such a difference [15].

In the body of the pancreas, our results show a reduction in the shear wave velocity as the BMI increases. Friedrich-Rust et al. found that the acoustic radiation force impulse imaging (ARFI) values in patients with pancreatic insufficiency due to cystic fibrosis were significantly lower than in those without insufficiency. The authors suggested that the deposition of fat in the tissues was responsible for this reduction [16]. A higher BMI and associated increase in total body fat means an increased risk of fatty deposition in the organs. Fat is softer than normal pancreatic parenchyma and its deposition may lead to the tissues becoming less firm, with a subsequent reduction in the shear wave velocity. Another possible explanation is the depth of the site where the measurements are being taken. Healthy volunteers with higher BMIs have a thicker abdominal wall, so that the organ lies further away from the transducer. This increased depth could result in lower measurements [29].

Our investigation into the effects of smoking, alcohol, and lipomatosis on the measurements revealed no significant relationships. It has to be remembered, however, that only a very small number of healthy volunteers were allocated to the positive control group in this random sample. Two studies have shown a significant reduction in the stiffness of the liver with transient elastography (TE) after abstinence from alcohol [35, 36]. The fact that we found no effects of alcohol consumption on our results may be partly due to the small number of healthy volunteers as well as to the way in which we recorded the history of alcohol consumption. We asked about the frequency of consumption rather than about absolute quantities of alcohol consumed and the duration of consumption.

Comparing subjects with pancreatic lipomatosis with those who did not have fatty infiltration of this organ did not produce any significant results. Motosugi et al. compared 121 patients with and without fatty deposition in the liver and 46 healthy volunteers without fatty organs. Here, too, there was no significant difference between the groups [37]. Although this result agrees with our current findings, it is somewhat unexpected, as lipomatosis should lead to softer tissue and hence lower shear wave velocities.

One of the limitations of the present study is the large number of examiners. The reliability measurements, however, show excellent results for the investigators involved in the study. Further, the angle and depth dependence of the method must be taken into consideration [38]. Also shear wave speed measurements can be influenced by increased subcutaneous fat. Since fat is soft, it can lead to a slow propagation of shear waves. This may lead to incorrect results. The pancreas lies in a close anatomical relationship with the aorta. Correspondingly, movement of the pancreas due to aortic pulsation affects the measurement of shear wave velocity. As it is impossible to eliminate this movement in living human subjects, its effects on the measurements remain unclear.

## Conclusions

In summary, we can say that our data confirm that age and gender influence the shear wave velocity of the pancreas. Future research with large-scale studies targeting healthy volunteers selected on the basis of their medical history is needed to address the questions of smoking, alcohol consumption, and pancreatic lipomatosis. A more precise history of alcohol consumption (quantity and duration) is required to determine the influence of alcohol. Other possible influencing factors, such as the pressure applied with the transducer, penetration depth, and precise phase of respiration, have also to be investigated in further studies.
